# Contrasting Fecal Methanogenic and Bacterial Profiles of Organic Dairy Cows Located in Northwest Washington Receiving Either a Mixed Diet of Pasture and TMR or Solely TMR

**DOI:** 10.3390/ani12202771

**Published:** 2022-10-14

**Authors:** Giovana Slanzon, William Sischo, Craig McConnel

**Affiliations:** Department of Veterinary Clinical Sciences, College of Veterinary Medicine, Washington State University, Pullman, WA 99164, USA

**Keywords:** archaea, 16S rRNA, organic dairy’ fecal microbiome, co-occurrence network analysis

## Abstract

**Simple Summary:**

Methane is a by-product of the microbial fermentation process from a group of archaea known as methanogens. Ruminants harbor methanogens as a component of their normal gastrointestinal microbiota. In this study, we assessed the fecal microbiome of organic dairy cows across different time points receiving a mixed diet of pasture and TMR or TMR only. Our aim was to compare the archaea and bacterial fecal microbial structure, diversity and their interactions across diets. Cluster analysis based on the cows’ fecal microbial community identified four clusters. We found little difference in the relative abundance of methanogens across clusters. However, there was evidence of differences in diversity between pasture associated communities and those associated with TMR only. The cluster associated with cows receiving high-quality pasture and TMR, had higher diversity and a less robust co-occurrence network than those in TMR only or lower-quality pasture communities. The overall good pasture and TMR quality, combined with the organic allowance for feeding high levels of TMR, might have contributed to the lack of differences in the fecal archaeal community. The development of novel strategies that are independent of pasture management could have a greater impact in helping to reduce enteric CH_4_ emissions on organic dairies.

**Abstract:**

Currently, little is known regarding fecal microbial populations and their associations with methanogenic archaea in pasture-based dairy cattle. In this study, we assessed the fecal microbiome of organic dairy cows across different time points receiving a mixed diet of pasture and total mixed ration (TMR) or TMR only. We hypothesized that the fecal methanogenic community, as well as co-occurrence patterns with bacteria, change across diets. To test these hypotheses, we analyzed TMR and pasture samples, as well as the V3-V4 region of 16S rRNA of fecal samples collected over the course of a one-year study period from 209 cows located on an organic dairy in Northwest Washington. The inherent variability in pasture quality, quantity, availability, and animal preference can lead to diverse dietary intakes. Therefore, we conducted a k-means clustering analysis to identify samples from cows that were associated with either a pasture-based diet or a solely TMR diet. A total of 4 clusters were identified. Clusters 1 and 3 were mainly associated with samples primarily collected from cows with access to pasture of varying quality and TMR, cluster 2 was formed by samples from cows receiving only TMR, and cluster 4 was a mix of samples from cows receiving high-quality pasture and TMR or TMR only. Interestingly, we found little difference in the relative abundance of methanogens between the community clusters. There was evidence of differences in diversity between pasture associated bacterial communities and those associated with TMR. Cluster 4 had higher diversity and a less robust co-occurrence network based on Spearman correlations than communities representing TMR only or lower-quality pasture samples. These findings indicate that varied bacterial communities are correlated with the metabolic characteristics of different diets. The overall good pasture and TMR quality in this study, combined with the organic allowance for feeding high levels of TMR even during the grazing season, might have contributed to the lack of differences in the fecal archaeal community from samples associated with a mixed pasture and TMR diet, and a TMR only diet. Mitigation strategies to decrease methane emissions such as increasing concentrate to forage ratio, decreasing pasture maturity and adopting grazing systems targeting high quality pasture have been shown to be efficient for pasture-based systems. However, the allowance for organic dairy producers to provide up to an average of 70% of a ruminant’s dry matter demand from dry matter fed (e.g., TMR), suggests that reducing enteric methane emissions may require the development of novel dietary strategies independent of pasture management.

## 1. Introduction

Methane (CH_4_) is a by-product of the microbial fermentation process from a group of archaea known collectively as methanogens. Ruminants harbor these methanogens as a component of their normal gastrointestinal microbiota. Methanogens metabolize several compounds including carbon dioxide (CO_2_), formate, methyl compounds, and acetate produced by other rumen microbes. The resulting metabolites combined with hydrogen (H_2_) result in the production of CH_4_ [1] [2]. Enteric CH_4_ produced during ruminal fermentation cannot be utilized by ruminants, being emitted into the atmosphere and contributes to greenhouse gas (GHG) emissions. In fact, enteric fermentation is the second largest contributor to CH_4_ emissions in the U.S. with the dairy sector accounting for 26% of the enteric emissions [3]. Developing a better understanding of emissions and designing dairy systems that mitigate CH_4_ is a challenge to the entire food animal production system.

Organic systems are rapidly becoming a key component of U.S. food animal production and are considered a model for sustainability [4]. Per the organic requirements for dairy management, cows are required to have free access to pastures throughout the grazing season and for no less than 120 days per year [5]. On average, at least 30% of a cow’s dry matter intake (DMI) during the pasture season must come from certified organic pasture. For that reason, the importance of pasture to organic dairy farming cannot be overstated. Organic production systems present a positive image to a segment of the public, [6] but relative CH_4_ emissions are reported to be higher on organic dairies as compared to conventional dairy farms due to lower milk production and increases in the intake of roughage [7,8]. Production of CH_4_ in pasture-based dairy systems is highly correlated to grass composition and digestibility. Variable diet compositions for dairy cows can impact CH_4_ emissions differently, due to the direct link to rumen fermentation patterns [9]. Despite the intrinsic complexity of host microbiomes across different sites, co-occurrent microorganisms were identified within the rumen and fecal microbiomes, indicating a strong association and inter-dependency between bacterial and archaeal communities of the same microbiome [10].

Factors associated with temporal changes in the external environment such as day length, air temperature, soil temperature, and water and nutrient availability can impact forage growth rate and accumulation of sugar (carbohydrates) and fiber (cell wall constituents) [11]. Therefore, pasture composition, quality, and availability are expected to differ with the seasonal changes to the external environment [12,13,14]. This will alter the diet of pasture-based organic dairy cows and is expected to impact the microbial community of the gastrointestinal tract [15]. In fact, seasonal changes in the diversity of rumen and fecal bacterial communities of grazing ruminants have been reported previously and linked to variations in pasture nutritive composition [16].

Three methanogenic archaea phyla and seven orders have been identified in ruminants [17]. The composition of the archaea community has a strong effect on CH_4_ emissions [18], and studies have demonstrated correlations between low and high CH_4_ emitting cows and their gastrointestinal tract microbiome composition [19,20,21,22]. *Methanobrevibacter*, from the order Methanobacteriales, have been identified as the most predominant and best represented CH_4_ producing methanogens in the rumen and feces of dairy cattle [23,24]. Changes to the diet can alter the methanogenic community structure and contribute to differences in CH_4_ gas production [24,25]. As an example, dry yeast additive has been reported to decrease CH_4_ production in vitro with associated changes to the fecal archaea including a decrease in *Methanobrevibacter* [24]. Currently, little is known regarding fecal microbial populations and their associations with methanogenic archaea in pasture-based dairy cattle. Therefore, we hypothesized that fecal methanogenic composition, as well as co-occurrence patterns with bacteria, would vary dependent upon dietary compositions of mixed pasture and total mixed ration (TMR). Moreover, we hypothesized that high- and low-quality pasture would impact the fecal microbial composition of organic dairy cows differently. To test these hypotheses, we analyzed fecal samples from 209 cows located on a Northwest Washington state organic dairy farm collected over the course of a one-year study period to capture changes to pasture composition and TMR intakes.

## 2. Materials and Methods

### 2.1. Study Design and Enrollment

This project was conducted on an organic dairy farm located in Northwest Washington state. The herd has been USDA-Organic certified since 2006 and housed approximately 700 lactating Holstein cows. Pasture and fecal samples were collected at 7 different time points between July 2020 and July 2021 to assess temporal changes in pasture quality and composition and their association with fecal microbiome community composition. At each sampling point, 30 primiparous healthy Holstein cows in the same housing group with access to the same pasture paddocks during the grazing months were enrolled in the study. Samples were collected from groups of cows representing different lactational stages (i.e., days in milk; DIM) to reflect different nutritional needs and feeding patterns that might contribute to changes in the microbial community and CH_4_ emissions. Within each group, cows had similar pregnancy status and milk production levels (±5 kg). Across the study, cows were only sampled once (i.e., there was no repeat sampling).

From approximately October through March (no pasture access), cows received a TMR consisting of a mix of soybean meal, alfalfa hay, mineral, grass silage, corn silage and corn grain. From April through September cows had access to TMR and a mixed grass and legume pasture targeting a dry matter intake of 30% pasture and 70% TMR. The grazing management used by this farm was strip grazing with irrigation as needed.

### 2.2. Data Collection

Pasture samples were collected one day prior to the collection of fecal samples to assess the pasture components and quality influencing the fecal microbiome analysis. We collected and analyzed 5 pasture samples per grazing paddock at each sampling time. The field was approximately 17 ha divided into 15 separated paddocks. Cows had access to a paddock from one up to three days depending on grass availability. Before collecting the pasture samples, 5 random measurements were taken to estimate the final grazing height. We estimated the final grazing height by following a “W” pattern in the paddock in which the cows were grazing previously. At each of the 5 “W” points, a 0.5 × 0.5 m^2^ quadrate made of plexiglass was thrown over the shoulder of the principal investigator to randomly place the quadrate within the paddock. A scale was used to estimate the final grazing heights. Next, pasture was sampled from the paddock in which the cows were grazing currently following the same “W” sampling pattern. Forage samples were collected using hand scissors and cutting to a level based on previous estimates of the final grazing height (6.3 cm on average). To estimate plant species diversity the percentage cover of grass, legume, and forb species within the quadrate was visually estimated [26]. Samples were stored in zip lock bags in a cooler with ice packs until they were delivered to the Ag Health Laboratories (Sunnyside, WA, USA), within 20 h after collection. Staff at Ag Health Laboratories prepared and processed samples for near infrared spectroscopy (NIRS) analysis.

NIRS also was used to analyze a representative sample of the TMR offered to the cows at each sampling time. TMR was mixed as per normal farm procedures, and we sampled immediately after delivery to the cows. We filled a five-gallon bucket with handfuls of TMR collected from the entire length of the feed bunk. The bucket contents were emptied on a flat surface and we collected samples from the top, middle and bottom to fill up a gallon zip lock bag. The bags were kept on a cooler with ice packs until they were delivered to the Ag Health Laboratories. During the 2 sampling months that cows were not utilizing pasture (October 2020 and March 2021), we only analyzed TMR samples. During July, September and October of 2020 we estimated dry matter intake (DMI) using the producers’ farm records and for March, May, June, and July 2021 we used the program “Onemilc” (Milc Group, San Luis Obispo, CA, USA) to gather the average DMI for these months.

Overall, we analyzed 25 pasture samples (July and September 2020 and May, June, and July 2021) and seven TMR samples (July, September, October 2020, and March, May, June, and July 2021). In addition, at each visit 30 fecal samples from 30 individual cows were collected and analyzed, except for July 2021, when there were only 29 cows eligible to be sampled for a total of 209 fecal samples collected and analyzed.

### 2.3. Amplification and Sequencing of Bacterial 16S rRNA Gene

Fecal samples were collected manually per rectum, placed in sterile sampling bags (Thermo Fisher Scientific, Waltham, MA, USA), and placed in a cooler with dry ice. On the same day, samples were transferred to Washington State University and stored in a −20 °C freezer until further processing within the Field Disease Investigation Unit laboratory. At the time of processing, fecal samples were thawed, mixed, and 1 g placed into DNA/RNA shield fecal collection tubes (Zymo Research, Irvine, CA, USA). DNA extraction and amplification of the V3–V4 region (primers 341F-806R) of the 16S rRNA gene was performed by Zymo Research (Irvine, CA, USA). The final library was sequenced by Zymo research using Illumina MiSeq. Unique amplicon sequences were inferred from raw reads using the dada2 pipeline [27]. Taxonomy assignment was performed using Uclust from Qiime v.1.9.1 [28].

### 2.4. Statistical Analysis

All statistical analyses were performed by the authors using R (R Project program for Statistical Computing 4.02).

### 2.5. Assessing Pasture Quality

Differences in pasture composition dry matter (DM), crude protein, acid detergent fiber (ADF), neutral detergent fiber (NDF), starch, crude fat, total digestible nutrients (TDN) and net energy for lactation (NEL) between sampling times were assessed using the aov function. If significant, pairwise comparisons were performed using the TukeyHSD function from the package stats. Since only a single TMR sample was collected at each sampling time, no statistical analyses were performed comparing TMR compositions between sampling times.

### 2.6. Evaluating Microbiome Community Structure

Beta diversity was analyzed using the ordinate function in R’s phyloseq package [29] to create a Principal Coordinates Analysis (PCoA) based on the number of sequence reads normalized by the number of total reads per sample and the modified Gower distance (altGower) [30]. A relative abundance heatmap was calculated based on the number of sequence reads normalized by the number of total reads per sample using the amp_heatmap function of the ampvis2 R package [31].

Cluster analysis was used to organize the individual microbiome data to create groups with similar microbiome community structures. Because there were many rare amplicon sequence variants (ASV) in the microbiome dataset we narrowed the dataset to reduce the average proportion of zero observed ASV to less than 0.20. This resulted in a dataset with 97 ASVs. The final dataset included four archaea ASVs for a total of 101 ASVs. Relative abundance of ASV by sample or animal ID was the initial input data for clustering. The clr transformation (centered log ratio) was performed using the R package compositions. Prior to implementing clr, zero values were imputed with non-zero values using R package zcompositions using a Bayesian multiplicative replacement. Principal component analysis (PCA) was used to reduce data dimensions with the R package prcomp. Prior to clustering we used the function fviz_nbclust from the R package factoextra as a guide in optimizing the number of clusters. Community clusters were calculated using the R stat package k-means as the cluster method using PCA results as input values and limiting the number of components to reflect approximately 90% of the variance. The final cluster solution was based on both silhouette plots and plots of the first 3 principal components stratified by cluster membership.

### 2.7. Assessing the Ecological Diversity of Fecal Microbiome by Cluster

A set of diversity measures converted to effective number of species were calculated to measure ecological diversity [32]. The mean of three commonly used measures, richness, Shannon index, and Simpson index, corresponding, respectively to q = 0, 1, and 2 were calculated for each of the clusters. The calculations were accomplished in R using the package vegan.

### 2.8. Fecal Microbiome Community Structure and its Associations with Pasture Intake, TMR Intake, and Pasture Quality

With cluster membership as the dependent variable, multinomial logistic regression was fitted to evaluate fecal microbiome community structure conditional on exposure to pasture and TMR or TMR only and community structure relative to pasture quality based on ADF and NDF. ADF and NDF were categorized as high or low using the median value. Analyses were done using the multinom function in R package nnet [33].

### 2.9. Co-Occurrence Network between Methanogens and Bacteria

A set of co-occurrence network analyses aiming to identify exclusive links between fecal methanogens and bacteria were determined using Spearman correlation coefficients at the species level. To test for differences in co-occurrence patterns in microbial taxa, we generated a matrix consisting of Spearman correlation coefficients based on a relative abundance table using the function rcorr from the Hmisc packge in R [34]. Mean relative abundance of species across clusters was calculated based on the average number of reads per species per cluster. The Spearman’s distance matrix represents the strength of correlation among microbial pairs (methanogens and bacteria); species were selected based on *p*-values (*p* < 0.05) and level of correlation (>60%). The function graph.adjacency from the R package iGraph [35] was used to create a graph object from the adjacency matrix. The nodes represented the different species, and the edge weight was based on the Spearman correlation.

## 3. Results

### 3.1. Sample Population

All sampled cows were first lactation cows of different lactational stages (early lactation: 20–80 DIM; mid-lactation 81–150 DIM, and late-lactation >150 DIM; *n* = 10 per group). The average DIM for early lactation cows was 47 (sd = 18 DIM), for mid lactation cows was 112 DIM (sd = 20 DIM), and for late lactation cows 206 DIM (sd = 45 DIM). Milk production and quality across different sampling times and cow’s lactation stages are reported in Table 1.

### 3.2. Feed Composition across Months

**Figure 1:**
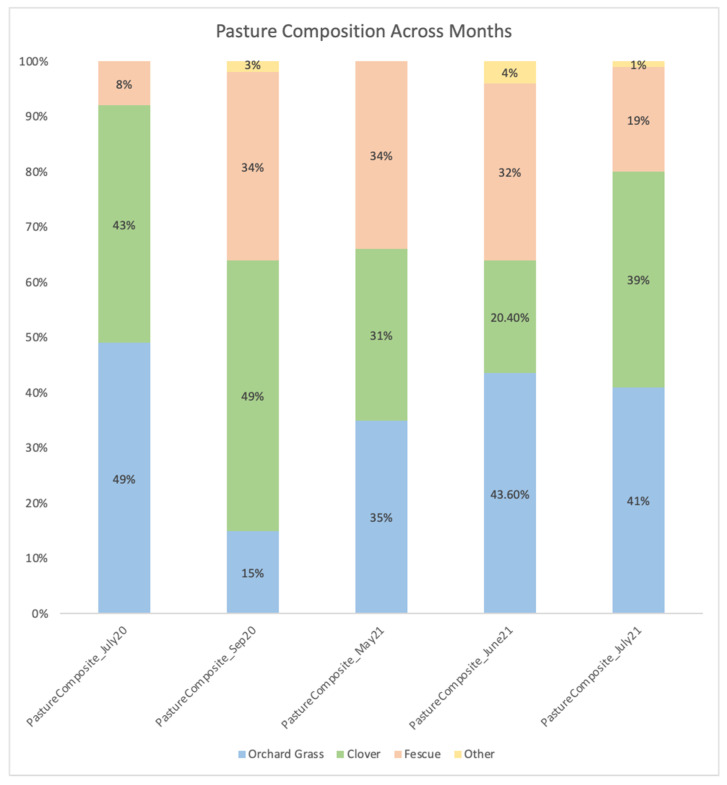
Pasture compositions across grazing months. The bars show the average percent of pasture species from 5 independent samples collected from the same paddock at a sampling period. Colors show the different pasture species.

Farm management planted pastures to be a mix of orchard grass, fescue, and clover with a minority of other plant species. The relative proportion of the plant species that the cows were grazing at the time of sampling varied across the grazing months (Figure 1). The two samples from 2020 were less balanced across the 3 primary pasture species relative to the 2021 samples. Both 2020 samples had greater than 40% clover with the July sample being composed primarily (>90%) of orchard grass and clover and the September being composed primarily (>80%) of fescue and clover. The May and June 2021 samples were balanced across the three species whereas the July 2021 sample tended to have a similar profile to the July 2020 sample although not as extreme with 80% of the species in the pasture being clover and orchard grass and nearly 20% being fescue.

NIRS analysis of pasture samples are shown in Table 2. Focusing on measures of digestibility, average ADF and NDF values were highest in July 2020, May 2021 and June 2021. Cows were not on pasture during October and March 2020 and those sample months were not included in this table.

Dry matter intake and NIRS analysis of TMR across sampling months is shown in Table 3. The TMR was not consistent across the sampling times and based on indicators of digestibility samples from March and June 2021 had the highest values for ADF and NDF whereas the sample from July 2021 had the lowest. These values are all consistent with a good quality forage.

### 3.3. Fecal Microbiota Structure across Different Sampling Points

**Figure 2:**
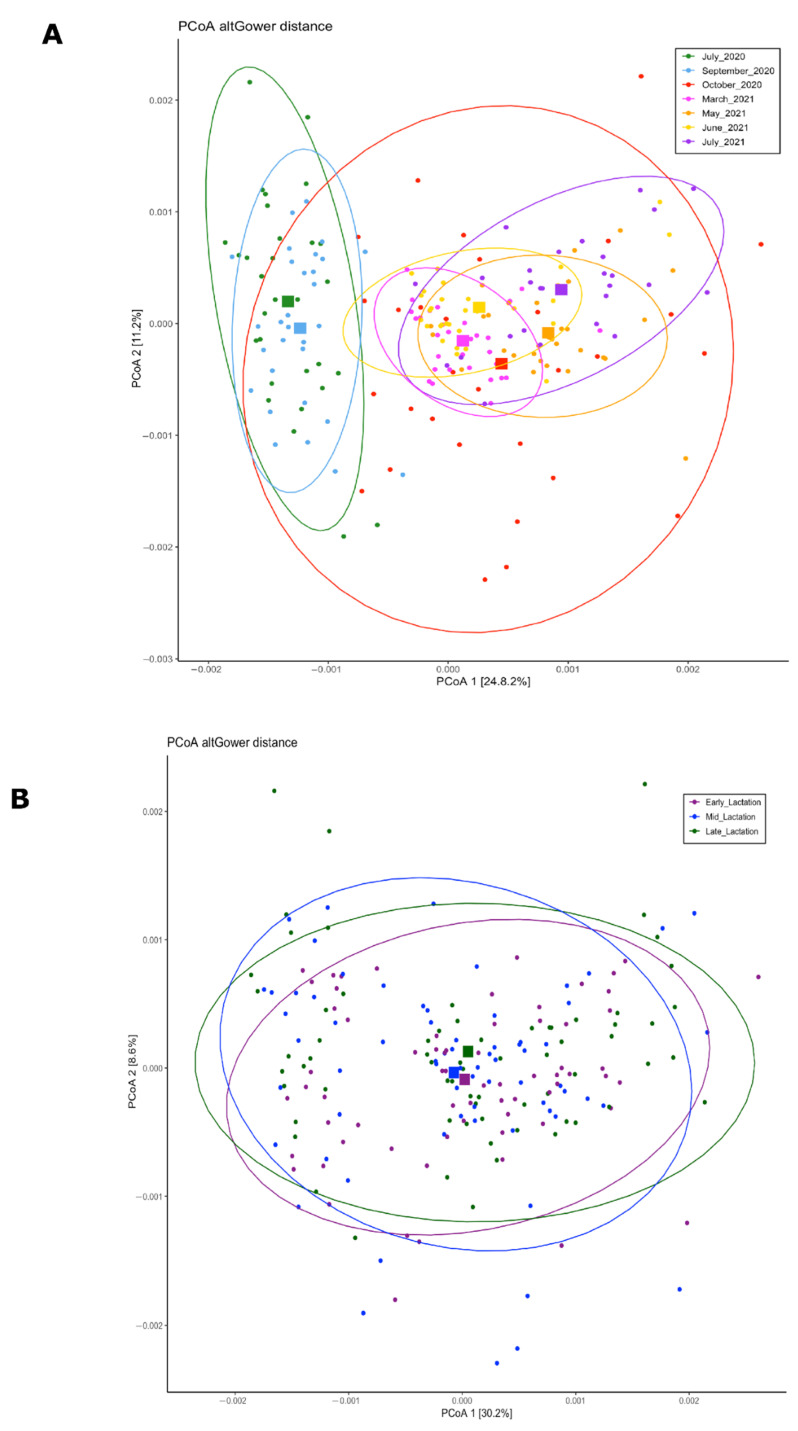
Principal coordinates analysis (PCoA) based on Gower distances. (**A**) PCoA grouped by sampling time. (**B**) PCoA grouped by lactation stage groups. Proportion of variance explained by each principal coordinate axis is denoted in the corresponding axis label. The different colors represent the different sampling points. Early lactation DIM = 20–80, mid-lactation DIM = 81–150, and late-lactation DIM > 150.

A total of 1715 unique microbial species, from a total of 203 different genera, were identified in the 209 fecal samples. A principal coordinate analysis (PCoA) based on Gower distances was performed to explore differences in the fecal microbiota composition at different sampling times and across different lactation stage groups (Figure 2). The results of the beta diversity analysis suggest a clustering of the microbial communities based on sampling date rather than lactation stage groups. Samples from 2020 pasture-exposed cows (July and September) grouped together, whereas the 2021 pasture-exposed samples also tended to group together.

### 3.4. Cluster Analysis

**Figure 3:**
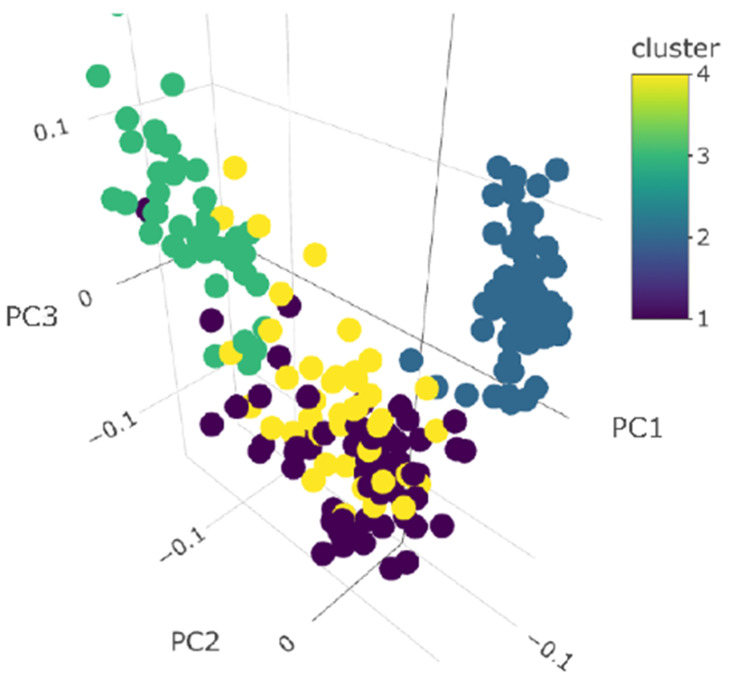
K-means clustering based on principal component values using relative abundance for 101 detected ASVs as the starting values. Different colors represent the different clusters.

Cluster analysis was used to describe microbiome community structure for the 97 most common ASVs and 4 detected archaea ASVs using all the collected samples. Input for clustering was based on principal coordinates values that reflected 82% of the cumulative variation of the complete set. The parsimonious cluster solution identified 4 clusters with an average silhouette width of 0.92 across all samples (Figure S1). The results of the analysis were plotted with 209 samples and the first 3 principal coordinates conditional on cluster membership (Figure 3). The number of animals present in each cluster according to their sampling time is shown in Table 4. Although all clusters contained samples from multiple sampling times, cluster 2 was dominated by samples collected in October 2020 (no pasture access) with only a single sample from March 2021 (no pasture access). The pasture-associated samples collected in 2020 were only found in cluster 3 whereas the pasture-associated samples collected in 2021 were mostly distributed in clusters 1 and 4. Although samples from a sampling period were distributed across various clusters, there was a tendency for samples at a sampling time to belong to a dominant cluster.

Categorizing samples based on access to pasture or a solely TMR diet relative to microbiome community cluster membership demonstrated sharp divisions. Samples in clusters 1 and 3 were primarily associated samples collected from cows with pasture access whereas samples in cluster 2 were solely associated with samples from cows without access to pasture. Cluster 4 contained samples collected from cows with and without access to pasture although it tended to be associated with samples from cows with access to pasture (Table 5).

### 3.5. The Relationship between Pasture ADF/NDF and Microbiome Community Structure

Pasture ADF and NDF were categorized into relatively high and low values using either the median value. For ADF, the median value was 29% of DM and for NDF the median value was 46% of DM. A frequency table of pasture ADF and cluster membership is shown in Table 6. Pasture NDF was perfectly correlated with pasture ADF. ADF categories were relatively evenly distributed across clusters 1 and 3. Cluster 4 mainly had pasture samples with high ADF.

Only samples of cows with access to pasture were taken into consideration in this analysis. Therefore, cluster 2 (solely TMR diet) was not taken into consideration.

The results of a multinomial logistic regression model with microbiome community clusters as the dependent variable (cluster 1 as reference) and ADF category as the independent variable are shown in Table 7. As noted above, cluster 4 was negatively associated with low ADF pastures compared to clusters 1 and 3.

### 3.6. Community Diversity

**Figure 4:**
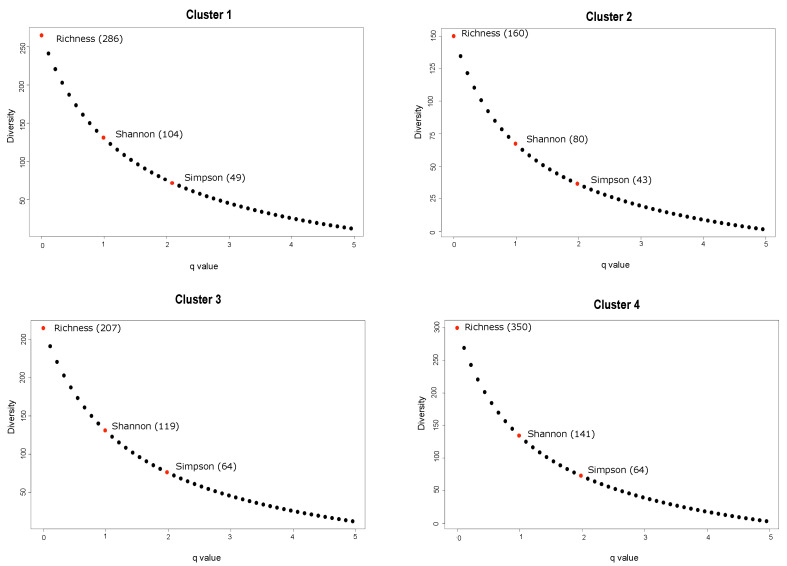
Effective number of species based on richness (q0), evenness (q1 = Shannon) and dominance (q2 = Simpson) across different clusters. The effective number of species are shown in parentheses.

Effective number of ASVs focused on diversity measures accounting for richness, evenness of taxa (based on Shannon index), and dominant taxa (based on Simpson index) present within the 4 clusters are shown in Figure 4. Differences in the effective number of observed species were observed across clusters ranging from 160–350 with cluster 4 having the greatest richness across all clusters while cluster 2 had the lowest number of observed species. The effective number of ASV reflecting evenness also differed between clusters and ranged from 80–141. Similar to species richness, cluster 4 had the greatest value based on the Shannon index, and the lowest value was observed in samples from cluster 2. The effective number of species reflecting dominance (Simpson index) ranged from 43–64 with clusters 3 and 4 having the greatest number, and clusters 1 and 2 having the fewest, 49 and 43, respectively. The general trend was clusters with samples from cows with access to pasture demonstrating increased diversity and the samples from cows with access to only TMR showing the lowest diversity.

**Figure 5:**
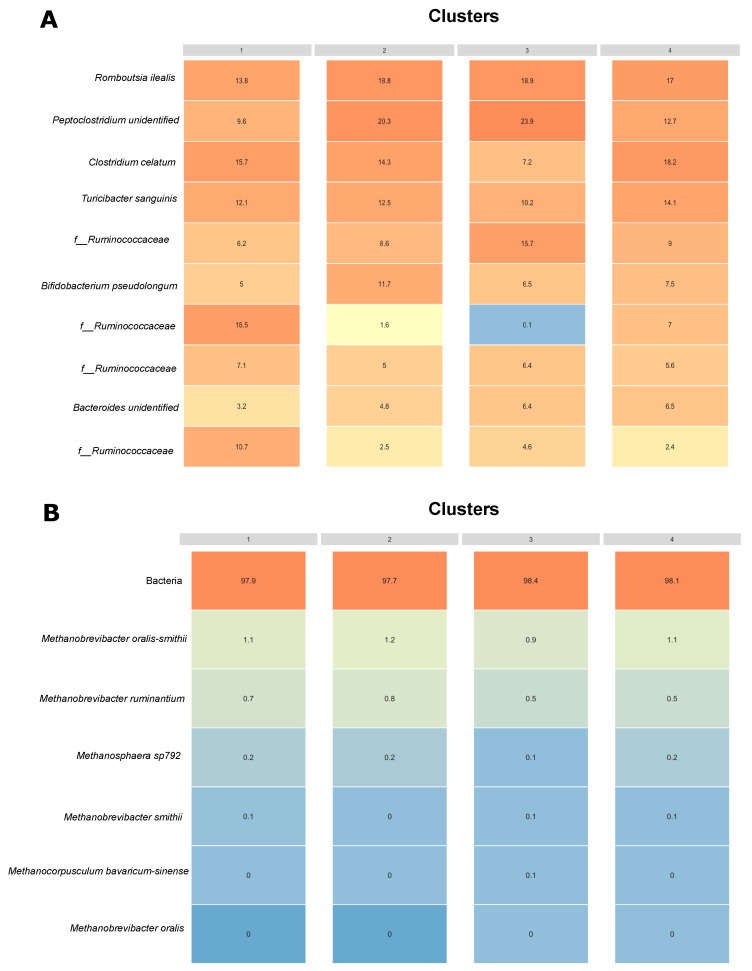
Relative abundance heatmap across clusters. Mean relative abundance for the most predominant bacterial species across clusters is indicated by the values in the tiles (**A**). Mean relative abundance of all archaea organisms at the species level across clusters is indicated by the values in the tiles (**B**). The color gradient indicates different levels in relative abundance.

A heatmap with the percentage of the empirical means of the relative abundance of bacteria (Figure 5A) and methanogens (Figure 5B) was calculated for cows across different clusters. Species previously classified as NAs were replaced with the next highest classification (e.g., if an ASV was unclassified at the genus level but was classified at the family level as “Ruminococcaceae”, it was given a family-level classification of “f_ Ruminococcaceae”). Overall, the relative abundance of methanogens was much lower as compared with the abundance of bacteria in the fecal microbiome of dairy cows. The Archaea community represented approximately 2% of the fecal microbial community when taking into consideration the two kingdoms. A total of six organisms belonging to the archaea kingdom were identified in our dataset: *Methanobrevibacter oralis-smithii*, *Methanobrevibacter ruminantium*, *Methanobrevibacter smithii*, *Methanocorpusculum bavaricum-sinense*, *Methanobrevibacter oralis*, and an unidentified organism of the genera *Methanosphaera*. As shown in Figure 5, no major differences were observed in the relative abundance of methanogens across clusters.

### 3.7. Co-Occurrence Network Analysis

**Figure 6:**
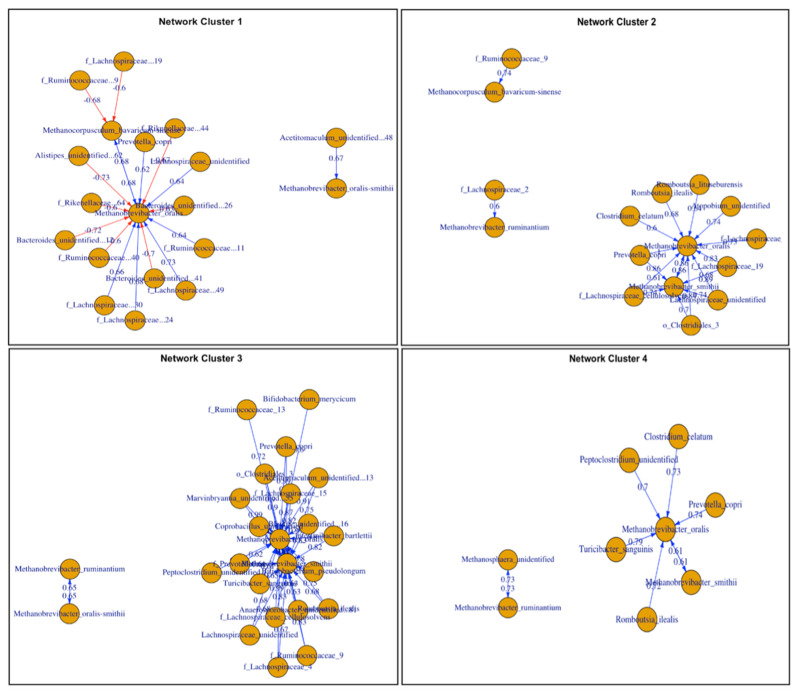
Co-occurrence network analysis based on Spearman correlation coefficients of bacterial species correlated with methanogens. The red color indicates a positive correlation and the blue color a negative correlation between the methanogens and the other bacterial species. ASVs were selected based on *p*-values (*p* < 0.05) at a 60% level of correlation with methanogens. Edge weight values are shown for each link between methanogens and bacteria.

To investigate whether there are differences in archaea-bacterial interactions in the gut microbial ecosystems across different diet compositions, the co-occurrence networks were analyzed exclusively for interactions between the two domains excluding bacterial-bacterial interactions. We created a co-occurrence network for each cluster (Figure 6). A total of 101 ASVs were compared based on Spearman correlation coefficients and were selected based on *p*-values (*p* < 0.05) at a 60% level of correlation. The edge weight was obtained based on the Spearman correlation, and we considered a negative co-occurrence relationship based on edge weight at values lower than r = −0.6 (red) and a positive correlation at values higher than r = 0.6 (blue). The co-occurrence network analysis results can be found at Table S1.

Based on Spearman correlations, samples associated exclusively with cluster 1 (Figure 6) had stronger negative correlations between archaea and bacteria compared with other clusters. Interestingly, we did not observe negative correlations at this specific cut-off for other clusters. Unidentified members of the family *Ruminococcaceae*, *Lachnospiraceae*, and *Rikenellaceae* as well as two unidentified ASVs of the genus *Bacteroides* and an unidentified species of the genus *Alistipes* were the specific bacteria negatively associated with the methanogens. Moreover, *Methanobrevibacter oralis* appeared to be the ASV with strongest and greatest number of co-occurrence interactions with the bacterial community regardless of the diet. Clusters associated with TMR only samples and a mix of pasture and TMR only samples (clusters 2 and 4, respectively) showed *Clostridium celatum* as an ASV strongly associated *Methanobrevibacter oralis* (rho = 0.6 and 0.73 for clusters 2 and 4, respectively). Moreover, *Methanobrevibacter oralis* was also positively and strongly correlated with *Methanobrevibacter smithii* in these clusters. Cluster 3, which was mainly composed of samples from cows with access to pasture, presented the strongest positive correlation values between bacteria and archaea. In this cluster, *Methanobrevibacter oralis* was strongly correlated (rho > 0.9) with unidentified species of the genus *Acetitomaculum*, *Coprobacillus*, *Marvinbryantia*, *Prevotella copri*, *Lachnospiraceae cellulosolvens*, and an unidentified species of the family *Ruminococcaceae.*

## 4. Discussion

The primary objective of this study was to evaluate the effect of mixed pasture and TMR dietary compositions of varying quantity and quality on the fecal methanogenic and bacterial composition from organic dairy cows located in NW Washington. Cows were sampled at 5 time points when they had access to both pasture and TMR targeting 30% DMI from pasture and 70% DMI from TMR. Cows were also sampled at 2 time points when they only had access to TMR. Bacterial and archaea communities were organized into 4 distinct clusters communities. Interestingly, there was little difference in relative abundance of methanogens between the community clusters; however, there was evidence of differences in diversity and structure between pasture associated communities and those associated with TMR. Community evaluations demonstrated that cluster 4 (associated with a high-quality pasture) had higher diversity and a less robust co-occurrence network than networks from TMR only or low-quality pasture communities.

Previous studies reported the use of NIRS as a method to estimate the chemical composition and nutritional quality of pastures and solid feeds being validated by wet chemistry results [36,37]. As expected, the botanical compositions, nutritional quality, and pasture digestibility levels were different across time points when analyzed by the NIRS method (Table 2 and Table 3). Fluctuations in quality throughout the grazing season can be related to changes in diversity of the grass-legume forage mix (Figure 1), forage maturity, climate, and overall pasture management across the year. A limitation of our project was the frequency of samples taken to describe changes in diet and microbiome across months. We were only able to procure individual snapshot assessments of pasture, TMR, and feces at infrequent time points throughout a year. However, these observations still allowed us to describe how changes in diet composition, as well as other intrinsic unknown factors associated with sampling time, can impact the fecal methanogenic and bacterial composition of pasture-based organic dairy cows. Although we collected fecal samples from cows during different lactational stages (DIM), we did not observe a clear separation between samples in the PCoA plot grouped by lactation stage, suggesting that the effect of diet and/or sampling time has a greater effect on fecal bacterial communities (Figure 2).

Per organic requirements, the farm aimed to feed a diet composed of 70% TMR and 30% pasture during periods of pasture availability. However, there was no specific mechanism in place to control the actual amount of pasture and TMR intake. Therefore, we could only estimate how much TMR was fed to the cows and the cow’s DMI across sampling months. Given the variability of pasture quality, quantity, availability, and animal preference, the estimated 70:30 ratio was likely different in practice and variable per individual animal. Therefore, we conducted a clustering analysis to identify samples that were associated with either a primarily pasture-based diet or a solely TMR diet. A total of 4 clusters were identified across all samples (Figure 3) and clusters were mostly formed by cows sampled at different sampling times (Table 4). The lack of clustering by sampling time might be explained by the different amounts of TMR and pasture consumed by each individual animal regardless of having access to pasture. A study evaluating the behavioral preferences of Holstein dairy cows indicated that cows had a partial preference to be indoors rather than on pasture, likely influenced by TMR consumption [38]. Other studies have suggested that dairy cows generally prefer to be on pasture; however, cows’ preference towards pasture or indoor housing depends on several factors such as the hour of the day, season, environmental conditions, and pasture distance [39,40,41]. Because we were unable to quantify the amount of TMR and pasture that was consumed by each individual, the cluster analysis allowed us to better characterize the associations between the fecal microbiome and diet.

According to the results of the clustering analysis, cluster 2 was highly correlated with a TMR diet whereas clusters 1 and 3 were associated with a more pasture-based diet (Table 4 and Table 5). Cluster 4 was characterized by a mix of samples from cows with access to pasture and TMR and a TMR only diet. Cluster 1 was composed of samples from May, June, and July of 2021 and cluster 3 was composed mostly by samples from July and September of 2020. In addition, according to the results of the multinomial logistic regression model, animals from cluster 4 that were exposed to pasture were associated with both high ADF and NDF (Table 6 and Table 7). Although the digestibility values were all consistent with a good quality forage, clusters were grouped into different pasture qualities to investigate how changes in pasture composition are associated with changes in the microbiome. It is possible that microbiome samples clustered together in cluster 4 (38 samples from cows with exposure to pasture and 28 samples from cows receiving TMR only) due to the high digestibility of diets offered.

Diversity analysis using different indexes aimed to explore the richness and evenness of the microbial communities across different clusters. A more diverse feed composition, such as the combination of pasture and TMR, is expected to give rise to a more diverse ruminal and fecal microbial community. Thus, samples from cluster 4, a mixture of exposures to pasture and TMR, had the greatest richness as well as effective number of species associated with evenness (Figure 4). This can be explained by the fact that cluster 4 contained multiple samples from diverse sampling points. On the other hand, samples from cluster 2, which only contained samples collected in October 2020 when cows were only receiving TMR, had the lowest richness as well as lowest effective number of species associated with evenness and dominance across clusters. The TMR fed to the cows was primarily composed of corn and grass silage. Preserved silage is characterized by low taxonomic diversity [42], as an intensive selection process occurs during the fermentation process and bacterial diversity dramatically declines as compared to the initial forage [43]. Moreover, changes over time in the silage bacterial profile, microbial diversity, and chemical traits [44,45] can influence a cow’s fecal microbiome. It also has been reported that feeding a highly grain-based diet reduces ruminal microbial richness and diversity compared to forage-based diets [46]. We observed the highest diversity based on Shannon and Simpson indexes from samples from cluster 3 and 4, suggesting a more uniform microbial distribution within these clusters. Dietary diversity has been suggested to influence ruminal microbial composition that consequently affects the animal’s wellbeing, health, dietary choices and dry matter intake [47].

The predominant bacterial species across all clusters included organisms from the genera *Clostridium*, *Rombustia*, *Turicibacter*, *Bacteroides*, *Bifidobacterium*, and unidentified species of the family *Ruminococcaceae* (Figure 5A). Members of these families have been previously reported to be part of the core rumen and fecal microbiome of cattle [2,48,49]. Methanogens accounted for approximately 2% of the overall relative abundance across all samples (Figure 5B). This observation is comparable to the abundance of archaea organisms observed in ruminal samples [50]. Differences in the relative abundance of methanogens across clusters associated with different diets were expected given that changes to the diet can alter the methanogenic community structure and contribute to differences in CH_4_ production [24,25]. However, we did not observe major differences in the relative abundance of methanogens across clusters. The relationship between CH_4_ production and the archaea community is so strict that a reduced archaeal abundance would be expected to lower CH_4_ emissions [51]. However, some studies have reported a poor correlation between the abundance of methanogens and CH_4_ emissions from dairy cows [20,21], suggesting that the methanogen community composition might have an outsized contribution to CH_4_ emissions. Overall, 6 species of methanogens were identified in our study. The species *Methanobrevibacter smithii-oralis* and *Methanobrevibacter ruminantium* were the most abundant methanogens in our dataset followed by an unclassified organism of the genus *Methanosphaera.* Our observations correlate with previous findings that *Methanobrevibacter* and *Methanosphaera* organisms dominate the ruminal methanogenic archaea community of cattle regardless of farming practices, geographical area or feed efficiency [2,25,52]. Even though we classified clusters based on their association with specific dietary composition and digestibility results, the pasture composition as well as the TMR values were all consistent with a good quality forage. Therefore, it possible that under good management practices, the diversity and abundance of fecal methanogens and potential enteric CH_4_ emissions from cows in organic systems do not differ from conventional dairy systems that also rely on a diet mainly composed of TMR.

The co-occurrence network analysis revealed different correlations between methanogens and bacteria across clusters associated with different diets (Figure 6). Based on Spearman correlations (*p* < 0.05; 60% level of correlation), samples associated with cluster 1 had stronger negative correlations between archaea and bacteria compared with other clusters. In addition, we did not observe other negative correlations at this specific cut-off in other clusters. The reasons for observing negative correlations at this specific cutoff only in cluster 1 remains unexplained; however, one limitation of our analysis is that the link between bacterial and archaea may not be straightforward, and might represent a complex relationship of interactions of diverse microorganisms such as fungi or protozoa that were not explored in the study. Although cluster 4 presented the highest diversity results, its network analysis showed fewer interactions between bacteria and archaea. This might be explained by the fact that samples from this cluster were associated with a highly digestible diet. Forage quality has been associated with changes in CH_4_ emissions, with lower levels of CH_4_ observed from cattle fed high quality as opposed to low quality forage [53,54]. Interestingly, only a few specific correlations were maintained across all the co-occurrence networks, suggesting that interactions can be specific to community structure and potential products present in the environment. However, *Methanobrevibacter oralis*, which has been previously identified as a colonizer of the human oral cavity [55], appeared to be the ASV with strongest and greatest number of co-occurrence interactions with the bacterial community regardless of the diet. The identification of keystone species that are maintained across different diets and farm management practices can contribute to development of new strategies to mitigate methane emissions.

It is a challenge to accurately compare the magnitude of the environmental impact of dairy systems under different feeding and management systems. GHG emissions arise from different biological and management processes in farm systems, which contributes to the difficulty in comparing the carbon footprint of pasture-based and confined dairies [56]. Different processes such as manure management, soil management, fertilization, and enteric fermentation are major contributors to the overall GHG emissions, with the latter being the largest source of GHG in dairy systems and the focus of our study [57]. Taken together, our results did not show major changes in the composition or relative abundance of methanogens across diets associated with a mixed pasture and TMR or TMR only diet. We also did not observe major differences in the relative abundance of methanogens across different pasture compositions based on digestibility results. However, we observed different co-occurrence patterns in the network analysis across different diets, suggesting that a varied bacterial community is correlated with the metabolic characteristics of different diets, resulting in different metabolites becoming accessible for methanogenic and bacterial activity and impacting the microbial interactions. The overall good pasture and TMR quality, combined with the organic allowance for feeding high levels of TMR even during the grazing season, might have contributed to the lack of differences in the fecal archaeal community of samples associated with a mixed pasture and TMR diet and a TMR solely diet. Given the complexity of assessing the environmental impacts of different systems, it is necessary to take into consideration other aspects of the production system. Carbon sequestration, external inputs, animal productivity, land area use, and social substantiality are important factors when comparing the overall impacts of organic and conventional systems. Moreover, mitigation strategies to decrease CH_4_ emissions such as increasing the concentrate to forage ratio, decreasing pasture maturity and adopting grazing systems targeting high quality pasture can be efficient for pasture-based systems. However, the allowance for organic dairy producers to provide up to an average of 70% of a ruminant’s dry matter demand from dry matter fed (e.g., TMR), suggests that reducing enteric methane emissions may require the development of novel dietary strategies independent of pasture management.

## 5. Conclusions

Variable diet compositions for dairy cows can impact CH_4_ emissions differently due to the direct link to rumen fermentation patterns and the available nutrients for microbial degradation. Therefore, extensive efforts have been made to manipulate the gut microbiome via dietary interventions or by utilizing CH_4_ inhibitors to reduce the environmental impacts of livestock. In this study, we did not observe major differences in the relative abundance or diversity of methanogens across clusters associated with a diet aiming to follow the organic requirements (30% pasture on a DMI basis) or a TMR only diet. This finding suggests that it possible that under good management practices, the diversity and abundance of fecal methanogens and potential enteric CH_4_ emissions of cows from organic systems do not differ from conventional dairy systems that also rely on a diet mainly composed of TMR. On the other hand, the co-occurrence network analysis revealed different correlations between methanogens and bacteria across clusters associated with different diets. Interestingly, *Methanobrevibacter oralis* appeared to be the ASV with the strongest and greatest number of positive co-occurrence interactions with the bacterial community regardless of the diet. The identification of keystone species that are maintained across different diets and farm management practices can contribute to the development of new strategies to mitigate CH_4_ emissions. Accurate associations between microbiome analyses and CH_4_ emission measurements are needed in order to utilize these outcomes and find effective microbial manipulation strategies or dietary interventions to help organic producers reduce enteric CH_4_ emissions. Mitigation strategies to decrease CH_4_ emissions such as increasing concentrate to forage ratio, decreasing pasture maturity and adopting grazing systems targeting high quality pasture can be efficient for pasture-based systems. However, since changing the concentrate to forage ratio is a limited strategy for organic producers, and the pasture portion required to be fed by the organic requirements is relatively small compared with the TMR portion, the development of novel strategies that are independent of pasture management could have a greater impact in helping to reduce enteric CH_4_ emissions through dietary manipulation on organic dairies.

## Figures and Tables

**Table 1:** Average milk production across lactation stages and sampling times and milk quality results across different sampling times.

	Milk Production (kg/day) by Lactation Stages			Milk Fat (%)	Milk Protein (%)
**Sampling time**	Early	Mid	Late		
**July 2020**	27.5	27.7	26.7	3.9	2.7
**September 2020**	21.5	27.3	25.6	4.1	3.0
**October 2020**	22.5	26.5	27.0	4.1	3.0
**March 2021**	27.6	29.2	24.7	4.0	3.2
**May 2021**	24.3	26.4	26.8	3.9	3.0
**June 2021**	25.6	27.9	25.5	4.1	3.1
**July 2021**	24.4	28.4	25.0	4.0	3.0

Results are based on producer records.

**Table 2:** Results of the NIRS analysis of pasture samples across different sampling times. The mean values for each pasture parameter were calculated and compared across sampling times.

	July 2020	Sep 2020	May 2021	June 2021	July 2021	Std. Error	*p*-Value
**DM (%)**	25.89 ^a^	19.17 ^bc^	17.08 ^c^	26.18 ^a^	23.86 ^ab^	2.04	0.0002
**Crude Protein**	15.52 ^b^	22.22 ^c^	20.03 ^ac^	17.04 ^ab^	17.97 ^ab^	1.29	0.0002
**ADF ^1^**	31.06 ^ab^	26.31 ^a^	29.05 ^ab^	30.28 ^ab^	26.38 ^a^	1.45	0.0067
**NDF ^2^**	45.95 ^a^	39.49 ^a^	46.5 ^a^	46.6 ^a^	40.18 ^a^	3.09	0.0540
**Starch**	3.41 ^a^	3.22 ^a^	2.08 ^b^	2.53 ^ab^	3.26 ^a^	0.37	0.0056
**Crude Fat**	3.31 ^c^	4.26 ^ab^	4.40 ^ab^	4.07 ^b^	4.50 ^a^	0.13	<0.0001
**TDN (%) ^3^**	62.87 ^c^	65.41 ^bc^	67.30 ^ab^	66.66 ^b^	70.55 ^a^	1.25	<0.0001
**NEL (Mcal/kg) ^4^**	1.29 ^c^	1.45 ^b^	1.49 ^ab^	1.49 ^ab^	1.60 ^a^	0.01	<0.0001

^1^ Acid detergent fiber. ^2^ Neutral detergent fiber. ^3^ Total digestible nitrogen. ^4^ Net energy of lactation. Crude protein, NDF, ADF, and starch are in DM%. Different letters (a, b, c) within a row denote significant differences by Tukey’s test (*p* < 0.05).

**Table 3:** Dry matter intake and NIRS analysis of TMR across sampling times. The TMR was composed of a mix of soybean meal, alfalfa hay, mineral, grass silage, corn silage and corn grain.

	July 2020	Sep 2020	Oct 2020	March 2021	May 2021	June 2021	July 2021
DMI *	16.3	16.3	19	19.1	15.1	15.3	19
**Dry matter (%)**	47.87	48.01	42.69	47.98	54.04	56.15	57.79
**Crude protein**	16.28	16.36	17.13	15.33	14.18	14.64	14.98
**NDF**	32.74	32.41	32.82	37.16	32.72	35.19	27.15
**ADF**	22.19	23.49	21.77	25.29	25.25	26.92	20.06
**Starch**	20.18	20.17	22.94	18.84	24.32	21.33	29.94
**Ash**	9.82	8.92	8.85	8.71	8.93	9.14	8.42
**TDN (%)**	70.03	68.96	70.87	67.51	69.29	68.23	72.58
**NEL (Mcal/kg)**	1.54	1.49	1.56	1.45	1.58	1.56	1.65

DMI = Average dry matter intake per cow/day (kg). Crude protein, NDF, ADF, starch and ash are in DM%. * Based on producer records.

**Table 4:** Number of fecal samples collected at each sampling period stratified by cluster membership.

	Sampling Time						
Clusters	July 2020	Sept 2020	Oct 2020	March 2021	May 2021	June 2021	July 2021
**1**	0	0	0	0	19	7	24
**2**	0	0	29	1	0	0	0
**3**	30	30	1	1	0	1	0
**4**	0	0	0	28	11	22	5

**Table 5:** Microbiome community membership relative to being fed solely a TMR or having access to pasture.

Microbiome Community (Clusters)	Access to Pasture (*n*)	No Access to Pasture—TMR Only (*n*)
**1**	50	0
**2**	0	30
**3**	61	2
**4**	38	28

**Table 6:** Frequency table of pasture ADF and NDF conditional on microbiome community clusters. ADF and NDF were categorized as high or low based on median values.

Microbiome Community (Clusters)	High Pasture ADF (>29%)	Low Pasture ADF (≤29%)	High Pasture NDF (>45%)	Low Pasture NDF (≤45%)
**1**	26	24	26	24
**3**	31	30	26	24
**4**	33	5	33	3

**Table 7:** Multinomial logistic regression evaluating the association between microbiome community and low pasture ADF.

Microbiome Community (Clusters)	*n*	Intercept	Odds Ratio	95% Lower Limit Confidence Interval	95% Upper Limit Confidence Interval
**1**	50	Reference			
**3**	61	0.17	0.05	−0.70	0.80
**4**	38	0.24	−1.80	−2.90	−0.71

## Data Availability

The data presented in this study are openly available in the NCBI database under BioProject accession number PRJNA881400.

## Supplementary Materials

The following are available online at https://www.mdpi.com/article/10.3390/ani12202771/s1, Figure S1: Silhouette plot result of cluster solution. Table S1: Co-occurrence network analysis results based on Spearman correlation coefficients of bacterial species correlated with methanogens.
