# Supplementary figures and images for: Contrasting Fecal Methanogenic and Bacterial Profiles of Organic Dairy Cows Located in Northwest Washington Receiving Either a Mixed Diet of Pasture and TMR or Solely TMR
Source: Animals (Basel). 2022 Oct 14;12(20):2771. doi: 10.3390/ani12202771 (PMC9597778; doi:10.3390/ani12202771)

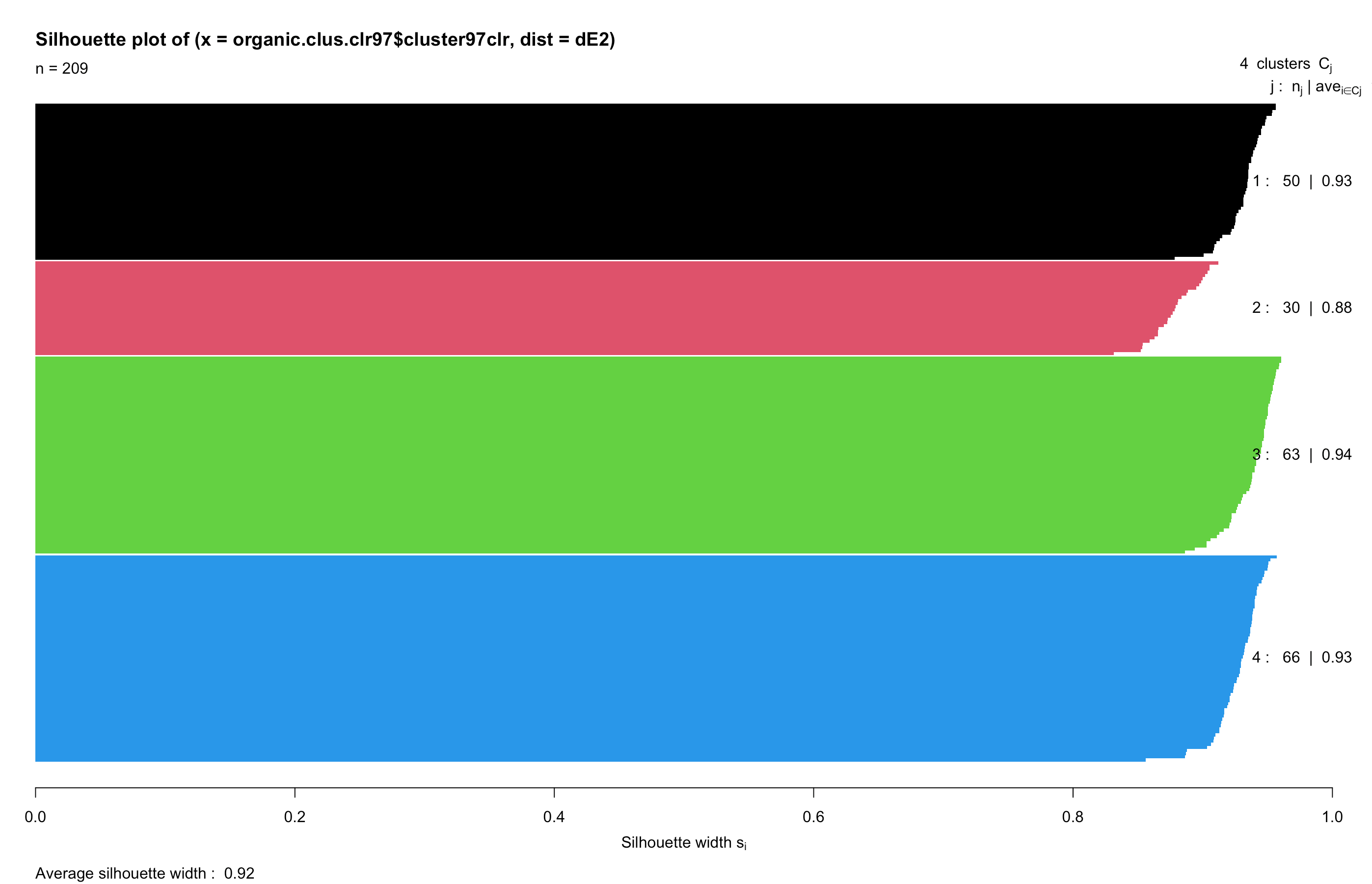

Supplement: Supplementary file 1 [file animals-12-02771-s001.zip › FigureS1.png]
